# Performance of the Linear Model Scattering of 2D Full Object with Limited Data

**DOI:** 10.3390/s22103868

**Published:** 2022-05-19

**Authors:** Ehsan Akbari Sekehravani, Giovanni Leone, Rocco Pierri

**Affiliations:** Department of Engineering, University of Campania “Luigi Vanvitelli”, I-81031 Aversa, Italy; ehsan.akbarisekehravani@unicampania.it (E.A.S.); rocco.pierri@unicampania.it (R.P.)

**Keywords:** linear inverse scattering, number of degrees of freedom, point spread function, resolution, aspect-limited excitations, aspect-limited observations, localization

## Abstract

Inverse scattering problems stand at the center of many important imaging applications, such as geophysical explorations, radar imaging, and synthetic-aperture radar (SAR). Several methods have been proposed to solve them when the full data are available, usually providing satisfactory reconstructions. However, it is impossible to acquire the full data in many practical circumstances, such as target detection and ground penetrating radar (GPR); consequently, only limited data are available. Thus, this paper focuses on the mathematical analysis and some numerical simulations to estimate the achievable resolution in reconstructing an object from the knowledge of the scattered far-field when only limited data are available, with multi-view excitations at a single frequency. We focus on 2D full rectangular geometry as the investigation domain (ID). We also examine the number of degrees of freedom (NDF) and evaluate the point spread function (PSF). In particular, the NDF of the considered geometry can be estimated analytically. An approximated closed-form evaluation of the PSF is recalled, discussed, and compared with the exact one. Moreover, receiving, transmission, and angle sensing modes are considered to apply the analysis to more realistic scenarios to highlight the difference between the corresponding NDF and the resulting resolution performances. Finally, interesting numerical applications of the resolution analysis for the localization of a collection of point-like scatterers are presented to illustrate how it matches the expectations.

## 1. Introduction

The inverse scattering problem is concerned with the reconstruction of the properties of an unknown object within a prefixed imaging domain from the knowledge of its scattered field, when incident plane waves illuminate it from multiple directions. When the Born approximation is invoked, it results in a linear inverse scattering problem. The inverse scattering problem arises in several areas in engineering, e.g., remote sensing [1,2], seismic imaging [3,4], radar imaging [5,6,7,8], synthetic-aperture radar (SAR) imaging [9,10], ground penetrating radar (GPR) [11,12,13,14], localizing thin metallic scatterers [15] and through-the-wall imaging (TWI) [16,17].

The complete knowledge of the entire angular domain of both plane wave incidence and scattered field observations is useful to determine the characteristics of an unknown scatterer completely; however, the full data are seldom available in applications. This means that the object is illuminated by plane waves impinging from a limited range of angles, and the scattered field is observed in a limited angular domain; this modality can be referred to as the aspect-limited excitations and observations case in the inverse scattering problem. Therefore, there is an enormous practical interest to recover the scatterer from the knowledge of the far-field pattern within a finite set of incidence and observation directions distributed over a limited range.

The limited view angles of incident plane waves and observations depend on the considered application; for instance, GPR requires finite view angles; conversely, the tomographic application usually takes into account full angle data.

The achievable resolution of the imaging algorithm, i.e., the minimum detail within the image that can be reconstructed, can be related to the point spread function (PSF). It provides the reconstruction of a point-like scatterer and depends on the amount of available scattered field data.

The number of independent pieces of information that can be reliably reconstructed by an imaging algorithm when noise is present in the data is provided by the number of degrees of freedom (NDF). The NDF has been used in [18] for optical imaging applications; furthermore, the NDF has been considered in inverse source problems for square [19] and circumference geometries [20] and inverse scattering problems [20,21,22]. The behavior of the PSF is likewise connected to the NDF of the problem.

The NDF of the relevant operator is provided by singular values decomposition (SVD). In fact, the NDF represents the number of significant singular values of the spectral decomposition of the relevant scattering operator; it may also be related in some way to the number of independent point-like scatterers that can be reconstructed accurately. In this way, it is also connected to the achievable resolution.

The resolution is defined in a radar system in terms of its ability to distinguish between two close objects, which is one of the most important criteria for assessing its efficiency. The concept of the PSF has been addressed in [23,24,25]. For instance, when the full data were available, we performed a similar PSF analysis in inverse source and scattering problems for the strip geometries in [26], where a good approximated PSF, the achievable resolution, and two applications were also provided. It was demonstrated that the resolution is the same regardless of the scatterer point position for the investigated geometries. In [20], the same investigation for circumference geometries is carried out.

In [27], localizing a slab embedded in a homogeneous half-space has been addressed for multifrequency; furthermore, it has been demonstrated that the maximum depth of investigation and the resolution limits depend on the properties of the investigated medium and on the work frequency band used.

The resolution and the physical reason for super-resolution in electromagnetic imaging for the half-space case have been studied in [28], using inverse scattering methods. A microwave tomography technique for imaging buried targets has been proposed in [29] under a forward-looking measurement configuration for the half-space 2D geometry; moreover, a theoretical analysis of the abilities of the achievable reconstruction has been provided for estimating the cross-range resolution limits.

A resolution analysis of the linear inverse scattering problem at a single frequency has been proposed in [20,26,30] by the estimation of the NDF of the relevant operator and the evaluation of the PSF in the object domain in the 2D scalar geometry. First, particularly in [20,26], for the case of round angle observation and impinging plane waves directions, curve investigation domain geometries (i.e., consisting of the boundaries of a 2D domain, as strips or arc of circumferences) were examined, since this allows one to evaluate in closed-form the NDF according to scattering object length. Instead, an approximate analytical expression of the PSF has been provided and numerically verified.

Next, the case of aspect-limited excitations and observations has been considered in [30] and applied to three different sensing modalities for the same geometries as in [20,26]. Then, the NDF cannot be computed in closed-form; however, a PSF analysis has been performed with the help of a different approximate analytical evaluation.

The novelty of the present paper is to be found in the evaluation of the achievable resolution for the 2D full rectangular geometry for impinging plane waves and far-field observations directions belonging to a limited range. In fact, it is important to examine the 2D full geometry to show the differences between the full and the void curve geometries of the previous papers. In this case, the NDF can be estimated in closed form; it means that the approximate PSF in the object domain is the same as in [30], since it depends only on the angular ranges of the available data in the aspect-limited configuration and not on the investigation domain geometry.

Again, for the sake of illustration, the receiving, transmission, and angle modes are recalled as in [30] to simulate the actual sensing configurations and to show the difference between their NDF and resolution. The main results to be mentioned are that they change when the range of plane waves and observation directions vary. Numerical simulations are presented to validate the estimation of the NDF, the analytical evaluation of the PSFs, and their role in the resolution. An application of the theoretical discussion for localizing scatterer points is proposed in the receiving and angle modes.

The rest of the paper is organized as follows. In Section 2, we discuss the NDF evaluation of the 2D rectangular geometry, recall the relevant approximated PSF, and, finally, validate it by some numerical simulations for the three sensing modalities. A numerical application of the theoretical discussions is provided in Section 3 by an illustrative localization problem. Section 4 contains a discussion and some concluding remarks.

## 2. Two-Dimensional (2D) Rectangular Geometry

**Figure 1:**
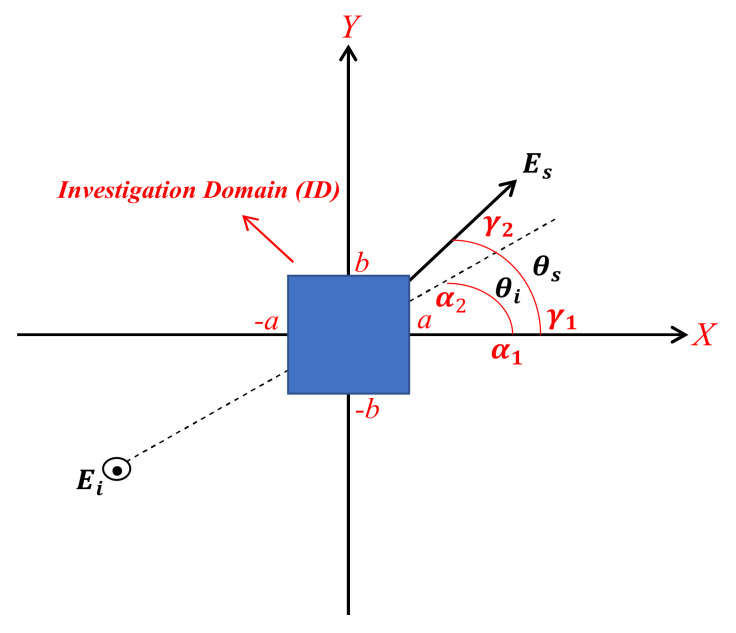
The geometry of a 2D rectangular.

This section addresses a two-dimensional (2D) rectangular investigation, as shown in Figure 1. Its length along the x and y is indicated by a and b, respectively. An unknown scatterer is located within this finite domain, i.e., the investigation domain (ID). We denote the angle providing the direction of propagation of the incident plane wave $E_{i}$ by $\theta_{i}$ and $E_{s}$ indicates the far-field response of the scatterer in the direction $\theta_{s}$. The plane waves and observations angular domains are supposed to be limited so that $\theta_{i}\in \left[\alpha_{1},\alpha_{2}\;\right]$ and $\theta_{s}\in \left[\gamma_{1},\gamma_{2}\;\right]$, respectively. The inverse scattering problem is stated as to determine the characteristics of the unknown scatterer from $E_{i}$ and $E_{s}$. It should be mentioned that the scattering sensing is investigated by using multiple plane waves with different incident angles to increase the NDF and to improve the reconstruction performance (multi-view configuration).

The relationship between the scattered field and the scatterer properties is nonlinear in general; however, under the Born approximation, the problem can be cast to a linear inverse problem. The linear model is only valid at a low wavenumber, where the scattered field is weak, but can also provide acceptable results in the case of metallic scatterers [31]. In the Cartesian coordinate system, the scattered far-field under the Born approximation from the 2D rectangular is expressed by

(1) $$E_{s}\left(\theta_{s},\theta_{i}\right)=\int_{-b}^{b}\int_{-a}^{a}\chi \left(x,y\right)\;e^{j\beta [x(\cos\theta_{s}-\cos\theta_{i})+y\left(\sin\theta_{s}-sin\theta_{i}\right)]}dxdy=T\left(\chi \left(x,y\right)\right)$$

where $\chi \left(x,y\right)$ and T are the contrast function and the linear operator for the multi-view and single frequency scattering configurations of our interest, respectively. Moreover, $x\in \left(-a,a\right)$ and $y\in \left(-b,b\right)$. It is supposed that the scatterer is located in a free space with a permittivity of $\epsilon_{0}$. Hence, $\chi \left(x,y\right)$ is equal to $1-\left(\epsilon_{s}\left(x,y\right)\right)⁄\epsilon_{0}$. Furthermore, the wavenumber and the wavelength are denoted by β=2π/λ and λ, respectively.

### 2.1. NDF Estimation

In order to estimate the NDF, we define spectral $k_{x}=\beta (\cos\theta_{s}-\cos\theta_{i})$ and $k_{y}=\beta (\sin\theta_{s}-\sin\theta_{i})$. As $\theta_{i}$ and $\theta_{s}$ span the $\left[\alpha_{1},\alpha_{2}\;\right]$ and $\left[\gamma_{1},\gamma_{2}\;\right]$ ranges, respectively, a spectral domain area denoted by A is spanned consequently. So (1) can be read as a double Fourier transform relationship.

(2) $$E_{s}\left(k_{x},k_{y}\right)=\int_{-b}^{b}\int_{-a}^{a}\chi \left(x,y\right)\;e^{j\beta \left[x\;k_{x}+y\;k_{y}\right]}dxdy$$

It should be noted that $\left(k_{x},k_{y}\right)\in A.$ Now, the NDF result of [32] can be recalled for a general 2D Fourier transform operator, as it is provided by $\Sigma \;A/{\left(2\pi \right)}^{2}$, where Σ is the measure of the area of the function to be transformed. For the considered ID, Σ is equal to 4ab.

**Figure 2:**
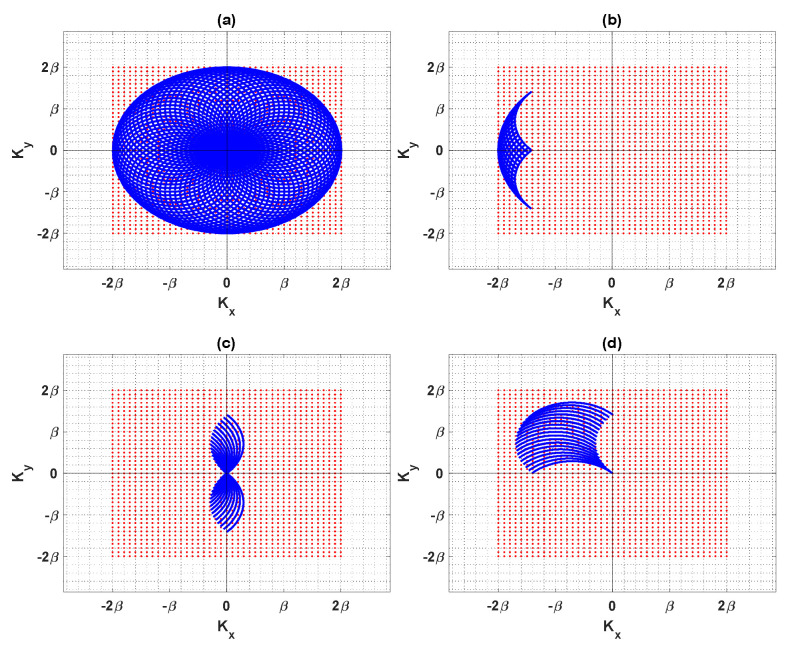
The domain of object function in the spectral domain: (**a**) full-view, (**b**) receiving mode, (**c**) transmission mode, (**d**) angle mode.

Accordingly, when the incident and observation angles change from 0 to 2π, the full object information in the spectral domain is achieved since, as shown in Figure 2a, the radius of the blue circle is 2β, its area will be $A=\pi {\left(2\beta \right)}^{2}$; thus, the NDF is equal to $4ab\pi {\left(2\beta \right)}^{2}/{\left(2\pi \right)}^{2}=16ab\pi \;/\lambda^{2}$.

For the sake of illustration, we recall three modalities from [30] to combine the impinging angles of the plane waves (transmitter) and observation directions (receiver) for the aspect-limited data case. First, it is supposed that the transmitters and receivers are located on the same side in the receiving mode, where the back-scattered fields are only available; it may be useful in GPR imaging and oil and gas exploration. Second, on the other hand, the transmission mode is specified when the receivers are on the opposite side of the transmitters, and the forward scattered fields are only available, for instance, in seismic prospection. Third, the angle mode is defined when the transmitters and the receivers are located on adjacent sides, which may occur in TWI applications. In particular, in order to highlight the reduction in the NDF for the aspect-limited case, we consider the same illustrative configurations as in [30], when the angular range of impinging direction is Δθ=π/2 wide and $\alpha_{1}$ and $\alpha_{2}$ are equal to −π/4 and π/4. Furthermore, the angular range of the observation directions is Δθ=π/2 wide, which changes from $\gamma_{1}=3\pi /4$ to $\gamma_{2}=5\pi /4$ when the sensing system is set in the receiving mode, from $\gamma_{1}=-\pi /4$ to $\gamma_{2}=\pi /4$ in the transmission mode and from $\gamma_{1}=\pi /4$ to $\gamma_{2}=3\pi /4$ in the angle mode, respectively.

Then, it is required to compute the spectral domain area for three considered modes to estimate the NDF. Figure 2b shows that the spectral domain area $A=\beta^{2}\left(\Delta \theta -\sin\Delta \theta \right)$ is small for the receiving mode, and the NDF is equal to $4ab\left(\Delta \theta -\sin\Delta \theta \right)/\lambda^{2}$. The NDF of the transmission mode is equal to $8ab\left(\Delta \theta -\sin\Delta \theta \right)/\lambda^{2}$ by calculating $A=2\beta^{2}\left(\Delta \theta -\sin\Delta \theta \right)$ in Figure 2c, which is double the NDF of the receiving mode. By computing the area $A=\beta^{2}(\pi -2\left(\Delta \theta -\sin\Delta \theta \right)$) in the angle mode, as observed in Figure 2d, the NDF will be $ab\left(4\pi -8\left(\Delta \theta -\sin\Delta \theta \right)\right)/\lambda^{2}$. Hence, the NDF is different for each mode because their spectral domain areas are not the same.

### 2.2. PSF Discussion

From a mathematical point of view, the PSF in the scatterer domain is defined as the (normalized) impulse response of the system, which is provided by the cascade of the $T^{-1}$, that is, the regularized inverse operator of T, and the direct operator, and was discussed in [20,26]. Unfortunately, it cannot be evaluated analytically for the scattering operator (1) as it requires the knowledge of its SVD. Moreover, since the regularized inverse operator depends on the NDF, it is important to determine it in closed-form in order to provide a correct reference behavior to be used for comparison purposes, when analytical results are not available. For the present aspect-limited configuration, this is not possible for curve geometries [30]; however, from the results of the previous subsection, this is made possible for the full 2D scatterer geometries.

As mentioned in the Introduction, the PSF knowledge can provide a quantitative tool to appreciate the resolution of the inversion algorithm, that is, the ability to reconstruct two close point-like scatterers. In fact, since it behaves like a sinc function, the width of its main lobe can provide a measure of this performance hereafter. In the Appendix A, for the sake of comparison, we recall the results for the full view case by resorting to the approximate $\tilde{PSF}$ as discussed in [20], since an analytical expression of the PSF is not available.

Now, following [30], it is also possible to consider an approximate $\tilde{PSF}$ for the present aspect-limited case as the product of the following four similar factors:

(3) $$\tilde{PSF}\left(x,x^{\prime },y,y^{\prime }\right)=\underset{n}{\sum }{\left(j\right)}^{n}J_{n}\left(\beta \left(x-x^{\prime }\right)\right)\;\left(\gamma_{2}-\gamma_{1}\right)\left(e^{\frac{1}{2}jn\left(\gamma_{2}+\gamma_{1}\right)}\right)\;sinc\;\left(\frac{n\left(\gamma_{2}-\gamma_{1}\right)}{2}\right)\;\;\underset{m}{\sum }{\left(-j\right)}^{m}\;J_{m}\left(\beta \left(x-x^{\prime }\right)\right)\;\left(\alpha_{2}-\alpha_{1}\right)\left(e^{-\frac{1}{2}jm\left(\alpha_{2}+\alpha_{1}\right)}\right)sinc\;\left(\frac{m\left(\alpha_{2}-\alpha_{1}\right)}{2}\right)\;\underset{\nu }{\sum }J_{\nu }\left(\beta \left(y-y^{\prime }\right)\right)\;\left(\gamma_{2}-\gamma_{1}\right)\left(e^{\frac{1}{2}j\nu \left(\gamma_{2}+\gamma_{1}\right)}\right)\;sinc\;\left(\frac{\nu \left(\gamma_{2}-\gamma_{1}\right)}{2}\right)\;\;\underset{\mu }{\sum }\;J_{\mu }\left(\beta \left(y-y^{\prime }\right)\right)\;\left(\alpha_{2}-\alpha_{1}\right)\left(e^{-\frac{1}{2}j\mu \left(\alpha_{2}+\alpha_{1}\right)}\right)\;sinc\;\left(\frac{\mu \left(\alpha_{2}-\alpha_{1}\right)}{2}\right)$$

where $\left(x,y\right)$ and $\left(x^{\prime },y^{\prime }\right)$ are the running and the scatterer point, respectively, and $J_{n}\left(.\right)$ is the Bessel function of the first kind and n-th order. In fact, the derivation of (3) does not depend on the ID, but only on the ranges of plane wave incidences and observation angles.

Equation (3) confirms that the $\tilde{PSF}$ is dependent on the distance between the source and the current points along each line, either vertical or horizontal. Accordingly, a constant resolution and spatial invariant behavior can be observed along each line. Nevertheless, the resolution along horizontal lines is different from the resolution along vertical lines. This allows predicting a space-variant behavior of the resolution of the reconstruction of a point-like scatterer, as will be verified in the following subsection.

### 2.3. Numerical Validation of the PSF Computation

We now discuss some numerical results to assess the performances of the results of the above-mentioned exact and analytical evaluations of the PSF. We assume that the considered scatterer is in a homogeneous space and a limited space problem. In particular, we consider a 2D rectangular dielectric with a=5λ and b=5λ, as shown in Figure 2. We employ a sufficiently adequate discretization of the relevant integral Equation (1) to compute the SVD of the resulting matrix equation under the MATLAB environment.

**Figure 3:**
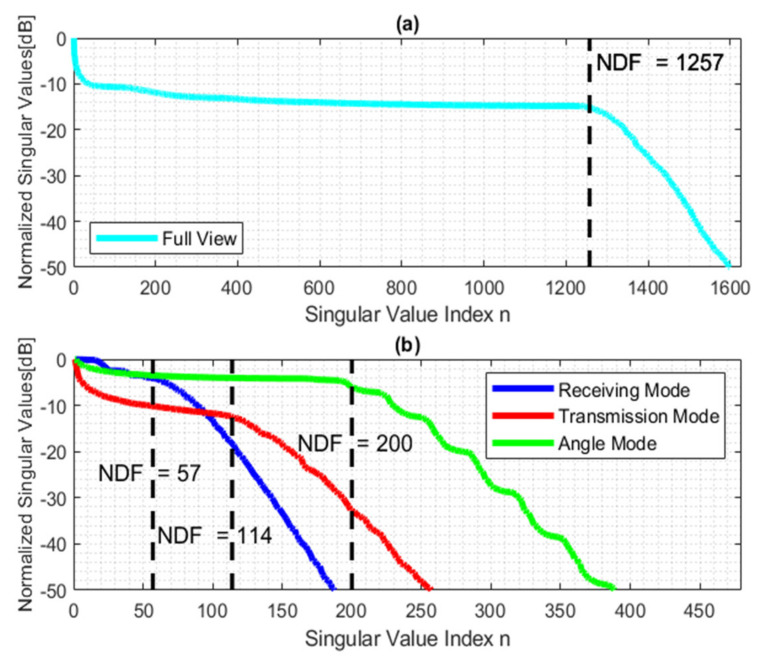
The behavior of the normalized singular values of the linearized inverse scattering: (**a**) full view, (**b**) three sensing modes. The dashed lack black line provides the analytically predicted NDF.

The aim of the first numerical simulation is to confirm the estimated NDFs for the full view case and the three modes at hand. Therefore, Figure 3 shows the pertinent behavior of normalized singular values of the relevant operator. It is worth noting that the singular values have a distinct step-like behavior from which the NDF can be evaluated. As predicted, the results confirm that the NDF is different for each mode in the aspect-limited case. In particular, Figure 3b confirms that the upper bound NDF of receiving, transmission, and angle modes is equal to 57, 114, and 200, respectively. It should be noted that the NDF of the transmission mode is twice the NDF of receiving mode; Figure 3a verifies that the NDF of the full view case is equal to 1257.

Therefore, to further evaluate the performance of the achievable resolution, a comparison between the actual PSF and the approximated one is provided to appreciate the accuracy of the approximated PSF for three modes. To this end, as a PSF has a typical sinc-like behavior, we only consider the main lobe of the PSF to outline the focusing properties and the amplitude of both PSFs is normalized to 1. The achievable resolution is generally estimated from the main lobe of the PSF by means of the full-width half maximum (FWHM) criterion, i.e., the resolution R is half of the width w of the main lobe of the function. We point out that it can be different in different directions according to the sensing modalities.

**Figure 4:**
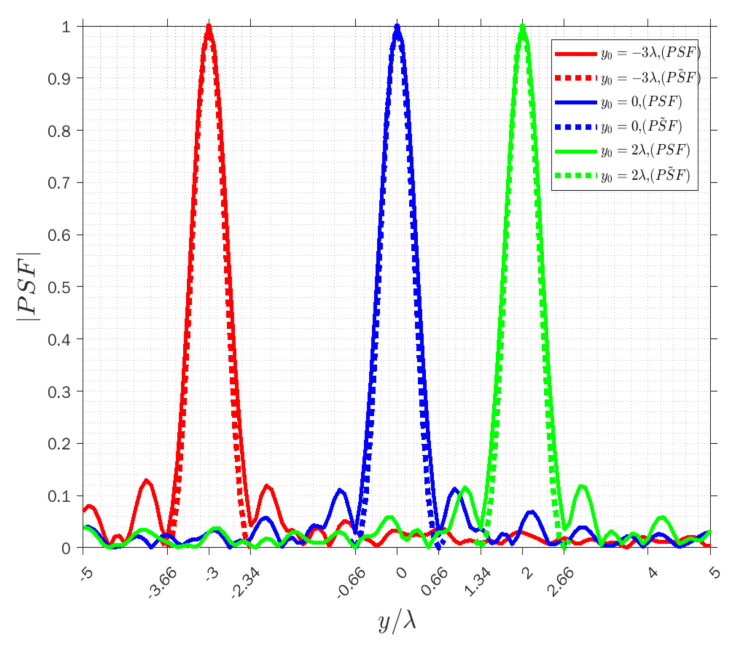
Comparison of the normalized amplitude of the actual (solid lines) and approximated (dashed lines) PSFs for $y_{0}=-3\lambda$ (red lines), $y_{0}=0$ (blue lines) and $y_{0}=2\lambda$ (green lines) when x=−λ in receiving mode.

**Figure 5:**
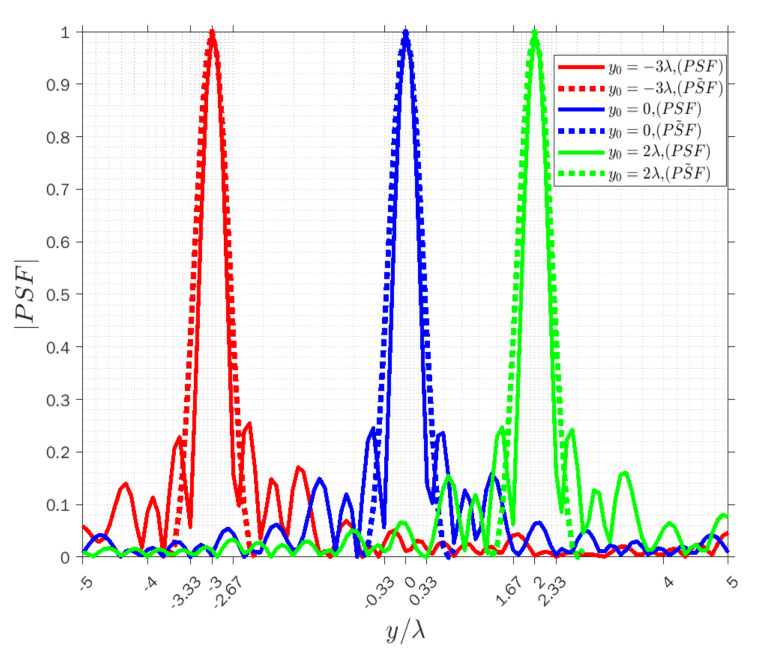
Comparison of the normalized amplitude of the actual (solid lines) and approximated (dashed lines) PSFs for $y_{0}=-3\lambda$ (red lines), $y_{0}=0$ (blue lines) and $y_{0}=2\lambda$ (green lines) when x=−λ in transmission mode.

**Figure 6:**
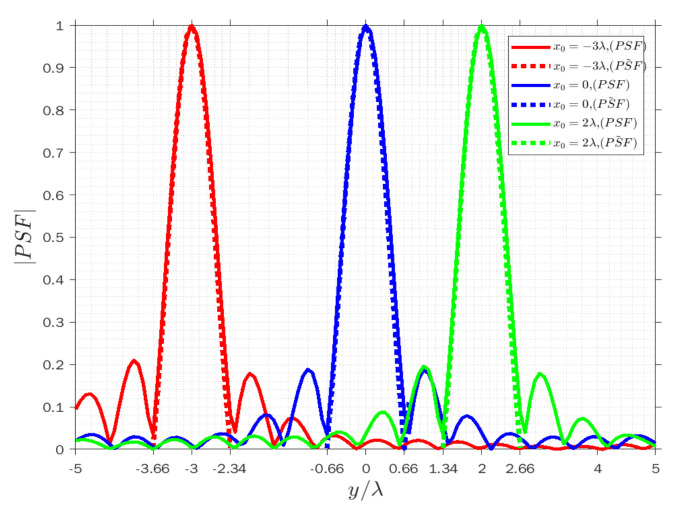
Comparison of the normalized amplitude of the actual (solid lines) and approximated (dashed lines) PSFs for $y_{0}=-3\lambda$ (red lines), $y_{0}=0$ (blue lines) and $y_{0}=2\lambda$ (green lines) when x=−λ in angle mode.

Figure 4, Figure 5 and Figure 6 display the normalized amplitude of both PSFs cut along y achieved when x=−λ for three modes. Specifically, Figure 4, Figure 5 and Figure 6 refer to the vertical y-cut for three different points. It is observed that the width of the main lobe and the resolution is $w_{vr}=w_{va}=1.32\lambda$, and $R_{vr}=R_{va}=0.66\lambda$ in the receiving and angle modes, respectively. On the other hand, the width of the main lobe and the resolution in the transmission mode is less than in the other modes, as they are equal to $w_{vt}=0.66\lambda$ and $R_{vt}=0.33\lambda$, as it can be predicted by the analytical behavior of (3). Furthermore, the width of the main lobes of both PSFs for three points is the same along with the cut in the three modes, which means the resolution is space invariant along the vertical lines. The results confirm that the approximated $\tilde{PSF}$ overlaps quite well with the actual PSF for each mode, which means that the approximation works satisfactorily in the main lobe in all three modes to predict the achievable resolution for every point scatterer.

**Figure 7:**
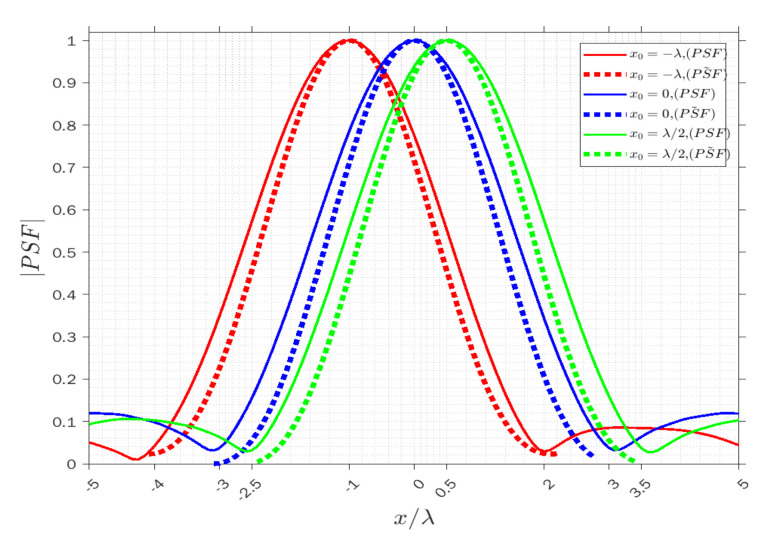
Comparison of the normalized amplitude of the actual (solid lines) and approximated (dashed lines) PSFs for $x_{0}=-\lambda$ (red lines), $x_{0}=0$ (blue lines) and $x_{0}=\lambda ⁄2$ (green lines) when y=0 in receiving mode.

**Figure 8:**
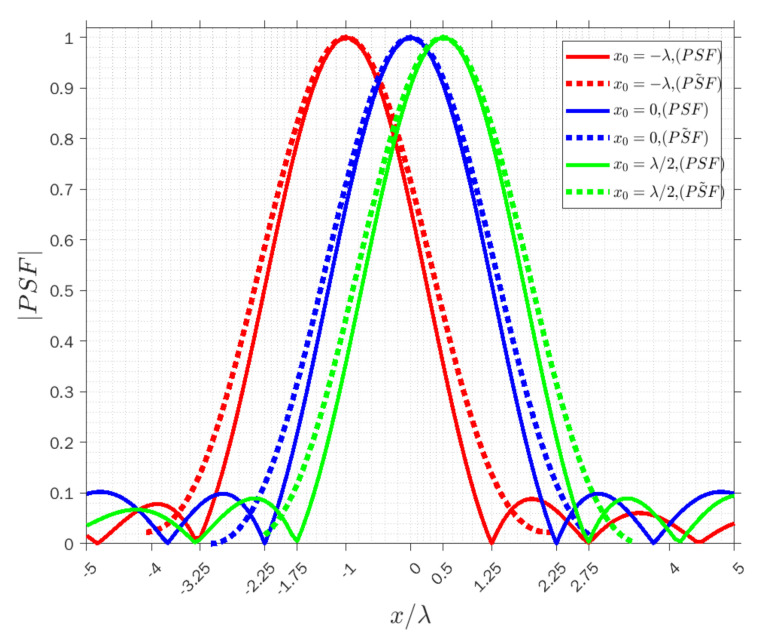
Comparison of the normalized amplitude of the actual (solid lines) and approximated (dashed lines) PSFs for $x_{0}=-\lambda$ (red lines), $x_{0}=0$ (blue lines) and $x_{0}=\lambda ⁄2$ (green lines) when y=0 in transmission mode.

**Figure 9:**
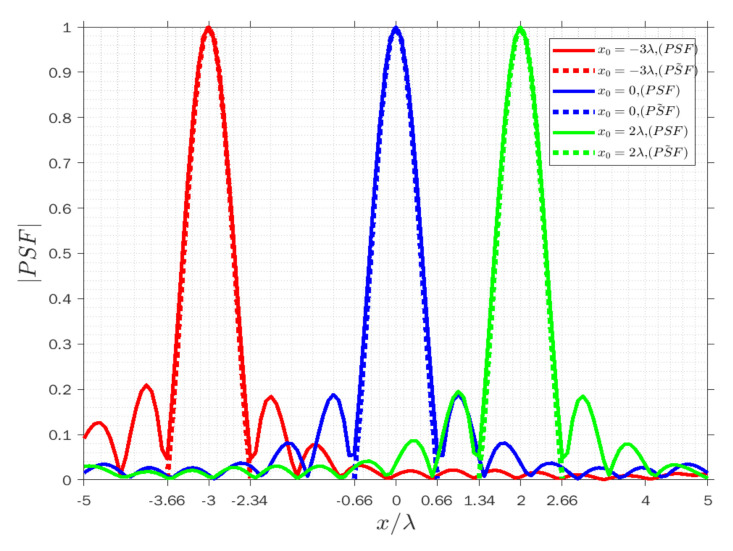
Comparison of the normalized amplitude of the actual (solid lines) and approximated (dashed lines) PSFs for $x_{0}=-\lambda$ (red lines), $x_{0}=0$ (blue lines) and $x_{0}=\lambda ⁄2$ (green lines) when y=0 in angle mode.

Similarly, Figure 7, Figure 8 and Figure 9 concern the horizontal x-cut of the actual PSF and approximated $\tilde{PSF}$ when y=0 in the three modes. It can be observed that the width of the main lobe and resolution is equal to $w_{hr}=6\lambda$, $R_{hr}=3\lambda$ in the receiving mode, $w_{ht}=4.5\lambda$, $R_{ht}=2.25\lambda$ in the transmission mode, and $w_{ha}=1.32\lambda$, $R_{ha}=0.66\lambda$ in the angle mode. The results clearly highlight that the resolution is again constant along x for each point and it is verified that the approximation approximately overlaps with the actual PSF at least in the main lobe, and it is acceptable in the three modes.

With regard to the vertical and horizontal resolutions, it turns out that the width of the main lobe of the PSF is different in the receiving and transmission modes; moreover, in the horizontal x-cut, the width of the main lobe is broader than the width of the main lobe in the vertical y-cut. It can be concluded that the space-variant behavior of the PSF only occurs when the scatterer point moves from one cut line to another one, and it depends on the range of transmitter and receiver directions. Nevertheless, we again observe that the PSF result is the same for both cuts in the angle mode, which means the resolution is unchanged on each cut, as it can be forecast by the analytical behavior of (3).

It should be noted that we also considered estimating the NDF and achievable resolution in curve geometries for the three modes in [30]. The results showed that the NDF of the three modes is approximately the same for the same angular range in the three modes, and the resolution changes as the position of the scatterer point varies. On the contrary, the NDF is different for each mode in 2D rectangular geometry; however, the resolution is not the same over the investigation domain in the receiving and transmission modes, and the resolution is always constant in the angle mode.

## 3. A Localization Application

This section provides a numerical application of the above theoretical discussions for the receiving and angle modes by numerically reconstructing a set of scattering points located within the ID in free space. The example can mimic object localization problems by multi-view multi-static radar systems with a finite number of independent transmitters and receivers. The background is air, and the contrast between the scatterers and the background is 1. The obtained results of Section 2 will also be used in this section.

**Figure 10:**
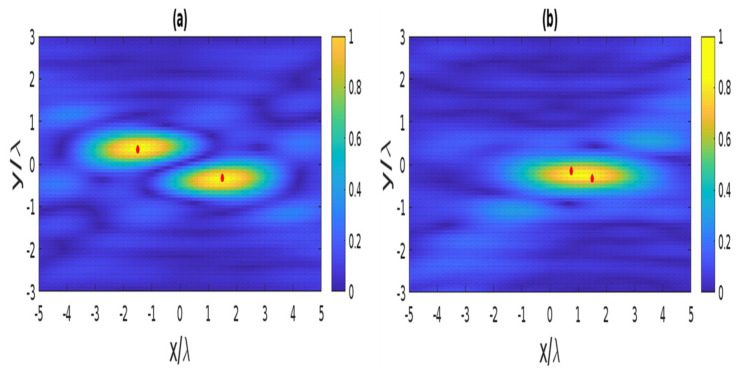
The reconstructed image of two point-like scatterers in the receiving mode when their spacing equals (**a**) and is less (**b**) the resolution. The red dots indicate the position of the scatterer points.

The first example concerns the reconstruction of two scatterer points in the receiving mode. Figure 10 presents the reconstructed images for different mutual distances. The amplitude of the PSFs is normalized to 1. As long as the distance between the two points is equal to the resolution, as predicted by (3), i.e., $R_{hr}=3\lambda$ and $R_{vr}=0.66\lambda$ are horizontal and vertical resolution, respectively; they can be perfectly resolved, as shown in Figure 10a. On the contrary, when the distance is equal to half of the resolution, it is impossible to localize two point-like scatterers; they appear as a single scatterer point, as shown in Figure 10b.

**Figure 11:**
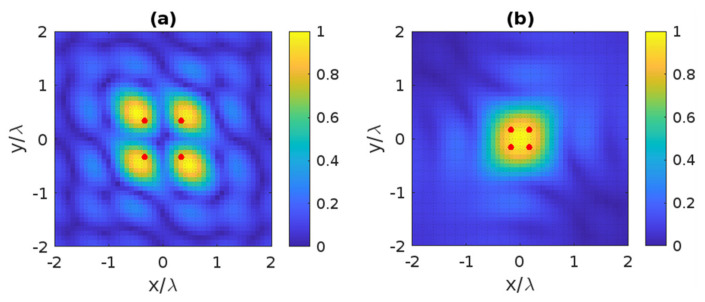
The reconstructed image of four point-like scatterers in the angle mode when their spacing equals (**a**) and is less (**b**) the resolution. The red dots indicate the position of the scatterer points.

The reconstruction of four scatterer points in the angle mode is considered in the second example. Now, the resolution is the same for horizontal and vertical lines, i.e., $R_{ha}=R_{va}=0.66\lambda$. Figure 11a illustrates the reconstructed images when the distance between two points is equal to the resolution; it is observed that the considered points can be entirely resolved. Then, four points appear as a single scatterer when the distance is equivalent to half of the resolution, as observed in Figure 11b.

**Figure 12:**
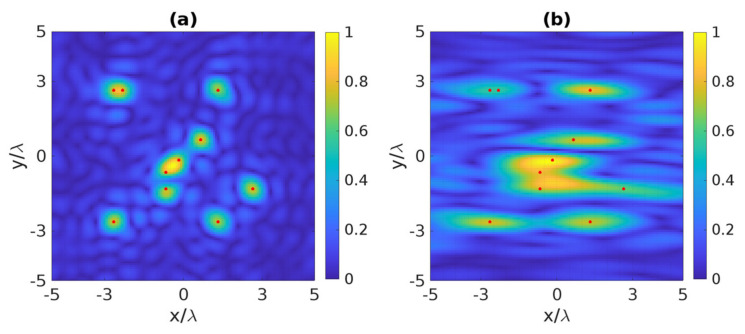
The normalized reconstructed image of ten point-like scatterers: (**a**) angle mode, (**b**) receiving mode. The red dots indicate the position of the scatterer points.

The third example is devoted to considering ten point-like scatterers that are randomly located within the ID for the angle and receiving modes, a rather challenging scattering scenario. In fact, the resolution is defined with reference to two point-like scatterers and the main lobe of the PSF. On the contrary, in the presence of a number of point-like scatterers, the presence of the side lobes of the PSF may affect the final reconstructed image, so to give rise to possible constructive and destructive interferences, with the other peaks corresponding to the scatterers’ location. This notwithstanding, the knowledge of the resolution according to the assumed sensing modality can also be helpful here. As can be observed in Figure 12a, when the distance between any couple of points is equal to or larger than $R_{ha}=R_{va}=0.66\lambda$, it is possible to localize correctly the scatterers; otherwise, they are unresolved in the angle mode.

Next, we change the sensing modality to the receiving mode and keep the previous ten point-like scatterers. As can be observed in Figure 12b, the two points can be differentiated when the distance between any couple of points is equal to or larger than $R_{hr}=3\lambda$ and $R_{vr}=0.66\lambda$, respectively; if not, they are indistinguishable in the receiving mode.

## 4. Discussion and Conclusions

The problems of the NDF and PSF of the linear inverse scattering have been addressed to estimate the achievable resolution for the 2D full rectangular geometry when the impinging plane waves and angular observation domains are limited. Therefore, the novelty of the manuscript relies on the extension of the validity of the approach introduced in [20,22] for curve geometries to a more general case. This also provides a larger scope for the applications, such as the localization problem considered in Section 3. The PSF can be evaluated by the numerical solution of the relevant inverse problem. Since the exact evaluation of the PSF can be accomplished only numerically for most geometries, an approximate analytical assessment, recalled from [30] for curve geometries, has been adopted and extended. Numerical simulations have demonstrated its accuracy against the actual PSF for the considered geometry.

As far as the NDF evaluation is concerned, at variance with [30] and where the NDF of the curve ID geometries has not been estimated theoretically as it is still an open problem, a closed form evaluation is now possible for the 2D full rectangular ID geometry in the present aspect-limited case. This information is useful to compute the reference PSF through the regularized inversion of the scattering operator.

For the sake of illustration, three different sensing modalities, i.e., the reflection, transmission, and angle modes, have been introduced to highlight their differences in NDF and resolution. In particular, the results show that the resolution changes as the position of the scatterer point varies along different lines parallel to the ID sides, but does not change along each line in the receiving and transmission modes. On the other hand, a near space invariant resolution is achieved over the whole geometry in the angle mode.

Finally, the resolution analysis has been applied to a numerical example concerning the localizing of a point-like scatterer from far-field data. The analysis can be useful to appreciate the expected performance of a multi-static multi-view surveillance radar imaging system according to the geometry of its transmitters and receivers and can be extended to the ID of a general 2D shape.

## Appendix A

For the sake of comparison, we report the results of the approximate closed-form evaluation of the PSF for the full view case and the resolution issue. In fact, if we start from (2) when A is a circle of radius 2β, then the adjoint of (2) reads as

(A1) $$\chi \left(x^{\prime },y^{\prime }\right)=\int_{}^{}\int_{A}^{}\;E_{s}\left(k_{x},k_{y}\right)e^{-j\beta \left[x^{\prime }\;k_{x}+y^{\prime }\;k_{y}\right]}\;dk_{x}\;dk_{y}$$

and the normalized $\tilde{PSF}$ is proportional to the integral

(A2) $$\int_{}^{}\int_{A}^{}\;E_{s}\left(k_{x},k_{y}\right)e^{j\beta \left[\left(x-x^{\prime }\right)\;k_{x}+\left(y-y^{\prime }\right)\;k_{y}\right]}\;dk_{x}\;dk_{y}$$

that can be evaluated in closed-form to provide the function

(A3) $$\tilde{PSF}\left(x,x^{\prime },y,y^{\prime }\right)=2\left[\frac{J_{1}\left(2\beta \sqrt{{\left(x-x^{\prime }\right)}^{2}+{\left(y-y^{\prime }\right)}^{2}}\right)}{2\beta \sqrt{{\left(x-x^{\prime }\right)}^{2}+{\left(y-y^{\prime }\right)}^{2}}}\right]$$

It can be verified that the corresponding resolution is R =0.3λ.
